# AgroEcoList 1.0: A checklist to improve reporting standards in ecological research in agriculture

**DOI:** 10.1371/journal.pone.0285478

**Published:** 2023-06-13

**Authors:** Georgia M. Daykin, Marcelo A. Aizen, Luke G. Barrett, Lewis J. Bartlett, Péter Batáry, Lucas A. Garibaldi, Ali Güncan, Sridhar Gutam, Bea Maas, Jayalakshmi Mitnala, Flavia Montaño-Centellas, Tarirai Muoni, Erik Öckinger, Ode Okechalu, Richard Ostler, Simon G. Potts, David C. Rose, Cairistiona F. E. Topp, Hope O. Usieta, Obaiya G. Utoblo, Christine Watson, Yi Zou, William J. Sutherland, Amelia S. C. Hood

**Affiliations:** 1 Department of Zoology, University of Cambridge, Cambridge, United Kingdom; 2 Instituto de Investigaciones en Biodiversidad y Medio Ambiente (INIBIOMA), Universidad Nacional del Comahue - Consejo Nacional de Investigaciones Científicas y Técnicas (CONICET), San Carlos de Bariloche, Río Negro, Argentina; 3 CSIRO Agriculture and Food, Canberra, ACT, Australia; 4 Center for the Ecology of Infectious Diseases, Odum School of Ecology, University of Georgia, Athens, Georgia, United States of America; 5 “Lendület” Landscape and Conservation Ecology, Institute of Ecology and Botany, Centre for Ecological Research, Vácrátót, Alkomány, Hungary; 6 Instituto de Investigaciones en Recursos Naturales, Agroecología y Desarrollo Rural, Universidad Nacional de Río Negro, Viedma, Río Negro, Argentina; 7 Consejo Nacional de Investigaciones Científicas y Técnicas, Instituto de Investigaciones en Recursos Naturales, Agroecología y Desarrollo Rural, Bariloche, Río Negro, Argentina; 8 Department of Plant Protection, Faculty of Agriculture, University of Ordu, Ordu, Turkey; 9 ICAR-AICRP on Fruits, ICAR-Indian Institute of Horticultural Research, Bengaluru, Karnataka, India; 10 Department of Botany and Biodiversity Research, University of Vienna, Vienna, Austria; 11 Agroecology, University of Goettingen, Göettingen, Germany; 12 Regional Agricultural Research Station, Acharya N. G. Ranga Agricultural University, Hyderabad, Andhra Pradesh, India; 13 Instituto de Ecología, Universidad Mayor de San Andrés, La Paz, Bolivia; 14 Department of Biological Sciences, Louisiana State University, Baton Rouge, Louisiana, United States of America; 15 CIMMYT Southern Africa Regional Office, Harare, Zimbabwe; 16 Department of Crop Production Ecology, Swedish University of Agricultural Sciences, Uppsala, Sweden; 17 Department of Ecology, Swedish University of Agricultural Sciences, Uppsala, Sweden; 18 Department of Plant Science and Biotechnology, University of Jos, Plateau, Nigeria; 19 Computational and Analytical Sciences, Rothamsted Research, Harpenden, United Kingdom; 20 Centre for Agri-environmental Research, School of Agriculture, Policy and Development, University of Reading, Reading, United Kingdom; 21 School of Water, Energy, and Environment, Cranfield University, Cranfield, United Kingdom; 22 Agriculture, Horticulture and Engineering Sciences, Scotland’s Rural College, Edinburgh, United Kingdom; 23 Leventis Foundation Nigeria, F. C. T. Abuja, Nigeria; 24 Rural Land Use, Scotland’s Rural College, Craibstone Estate, Aberdeen, United Kingdom; 25 Department of Health and Environmental Sciences, Xi’an Jiaotong-Liverpool University, Suzhou, P. R. China; Instituto Federal de Educacao Ciencia e Tecnologia Goiano - Campus Urutai, BRAZIL

## Abstract

Many publications lack sufficient background information (e.g. location) to be interpreted, replicated, or reused for synthesis. This impedes scientific progress and the application of science to practice. Reporting guidelines (e.g. checklists) improve reporting standards. They have been widely taken up in the medical sciences, but not in ecological and agricultural research. Here, we use a community-centred approach to develop a reporting checklist (AgroEcoList 1.0) through surveys and workshops with 23 experts and the wider agroecological community. To put AgroEcoList in context, we also assessed the agroecological community’s perception of reporting standards in agroecology. A total of 345 researchers, reviewers, and editors, responded to our survey. Although only 32% of respondents had prior knowledge of reporting guidelines, 76% of those that had said guidelines improved reporting standards. Overall, respondents agreed on the need of AgroEcolist 1.0; only 24% of respondents had used reporting guidelines before, but 78% indicated they would use AgroEcoList 1.0. We updated AgroecoList 1.0 based on respondents’ feedback and user-testing. AgroecoList 1.0 consists of 42 variables in seven groups: experimental/sampling set-up, study site, soil, livestock management, crop and grassland management, outputs, and finances. It is presented here, and is also available on github (https://github.com/AgroecoList/Agroecolist). AgroEcoList 1.0 can serve as a guide for authors, reviewers, and editors to improve reporting standards in agricultural ecology. Our community-centred approach is a replicable method that could be adapted to develop reporting checklists in other fields. Reporting guidelines such as AgroEcoList can improve reporting standards and therefore the application of research to practice, and we recommend that they are adopted more widely in agriculture and ecology.

## Introduction

*Reporting standards* refers to the completeness and accuracy with which the methods, relevant metadata and results of a study are reported; higher reporting standards enable studies to be understood, replicated, and reused effectively (e.g. for meta-analyses), both by scientists and wider stakeholders [1–3]. Better reporting facilitates the uptake of research findings and recommendations by practitioners and policy-makers, something which is currently lacking [4]. By improving the description of the context within which the research was conducted (e.g. the soil type that the study was conducted in), practitioners can better evaluate the relevance of research to their specific situation. Better reporting standards can also benefit individual researchers as publications with more complete reporting are more cited [5]. Despite this, current reporting standards are often poor, and basic details are regularly missing from reports [6]. For instance, a review of on-farm experimental studies found that important details such as field history, land preparation and management of pests and water were rarely reported [7]. An analysis of 160 publications on freshwater ecology found a lack of information essential for replication, with some fundamental variables, such as exact study dates and distance between samples, reported in <40% of articles [8]. A systematic review of agricultural management on soil carbon found that 70 of 500 studies did not report the experimental design (e.g. whether it was randomised, split-plot, etc.) [9]. Finally, a systematic map of 190 studies on the impacts of farmland abandonment found that 105 studies did not report the experimental spatial scale, 40 did not report the intervention duration, 38 did not report the intervention timing, and 28 did not report the number of replicates [10]. Poor reporting causes research wastage and means that the “Interoperable” and “Reusable” aspects of the are not being met [11]. The consequences of poor reporting are increasingly recognised; 52% of researchers agree that there is a “significant crisis of reproducibility” in science [12], and calls to address this have been made in agricultural and ecological research [7, 13].

*Reporting guidelines*, in the form of checklists, text or diagrams, outline information that should be reported to improve reporting standards [11]. In particular, checklists can increase the collection of “zero” data (i.e. they encourage reporting practices that weren’t done, e.g. “no fertiliser was applied”). Over the last two decades, many reporting guidelines have been developed and applied in the field of medicine [14], and these guidelines have successfully improved reporting standards [15–19]. For example, Plint *et al*. (2006) found that studies that used the CONSORT checklist (CONsolidated Standards of Reporting Trials) included significantly more methodological details than those that did not [16]. Similarly, publications in journals endorsing the use of the CONSORT or PRISMA (Preferred Reporting Items for Systematic Reviews and Meta-analyses) checklists have higher reporting standards than those from non-endorsing journals [15, 17, 18]. Many medical journals now endorse reporting guidelines as a result [20], and medical reporting guidelines have been consolidated into an online website–The EQUATOR Network [21]–where users can easily find relevant guidance.

Reporting standards and guidelines have received less attention in agricultural and ecological research, but interest is growing [6, 22]. Some agricultural or ecological journals direct authors and reviewers to some of the relevant reporting guidelines (e.g. the *International Journal of Ecology* (Published by *Hindawi)* [23], *Agricultural Research (Springer)* [24], and the *Journal of the Science of Food and Agriculture (Wiley)* [25], but there is no consolidated repository where users can easily find the relevant guidelines (e.g. the Equator Network for the medical sciences). The relevant checklists include: agricultural microbiome research [26], agricultural nutrient management research [27], arthropod abundance data [28], ecological datasets [29], evidence-synthesis in ecology and evolutionary biology [19, 30], genome sequencing [31], plant phenomics [32], and transparency in ecology and evolution [33]. *Nature Publishing Group* [34] and *Ecology Letters (Wiley)* [35] have provided their own reporting checklists in author guidelines for experimental details. These checklists are topic-specific, and many knowledge gaps remain.

Standardised metadata formats for datasets are similar to reporting checklists (and can overlap), but they differ in several ways. *Standardised metadata formats* for datasets aim to standardise the way that data is reported (e.g. variable names and units) so that it can be rapidly (even automatically) extracted [36]. To do this, complex multi-level ontologies are developed [36, 37], and often maintained as living documents online [38–40]. Multi-stakeholder groups have developed ontologies for metadata in agriculture and ecology, some of the most relevant of which are: ICASA Data Standards for data from agricultural field experiments [41, 42]; DarwinCore for taxonomic data [38]; AgrO for agronomic data [40] and the Plant Experimental Conditions Ontology for plant biology experiment [43]. AgroPortal has collated the ontologies relevant to agronomy (n = 142) online [36] and an open-source online tool (Agronomy Field Information Management System (AgroFIMS)) has been developed to facilitate their use [44]. We recommend that researchers use these ontologies and tools to make their research data more interoperable and reusable [11]. Reporting checklists can complement these ontologies as the relevant standardised metadata formats are often for “maximal datasets” (i.e. they list most variables that may be reported rather than a minimum number of variables that should be reported) [40, 41]. Reporting checklists also ensure the most relevant information is reported within the text, which makes it accessible to readers who do not have time to delve into the datasets. Raw data can be difficult to understand and misunderstood, especially if time is limited [22]. They also ensure that the most important information is reported if the researchers do not provide the data. However we strongly recommend that authors do provide their data as there are many benefits of doing so, both to authors and the wider scientific community [45].

Here, we fill an important gap in reporting checklists by taking a community-centred approach to co-develop a reporting checklist (AgroEcoList 1.0) for ecological studies in agriculture. We aim to improve reporting standards following a similar strategy to that which has proved successful in medical research [15–17] AgroEcoList captures the key variables that authors, reviewers, and editors should consider reporting when planning, drafting, reviewing, or editing studies. It should be used in tandem with standardised metadata formats for datasets. To put AgroEcoList in context and test its usability, we also assess the agroecological community’s perception of reporting standards and guidelines in agroecology.

## Materials and methods

### AgroEcoList experts

A group of 23 experts (co-authors) was assembled in June-July 2021. We used a survey to guide this process (questions provided in full in S1 in S1 File) following approval from the University of Reading’s ethics committee [reference number: 1663D]. The survey gathered information on the team’s career stages in academia, fields of expertise, countries of affiliation, and genders. We continually assessed the diversity and expertise of the group using these parameters, and recruited new members to increase diversity and representation of participants while keeping it a manageable size. Recruitment was conducted using existing networks, collaborators and via social media.

**Fig 1:**
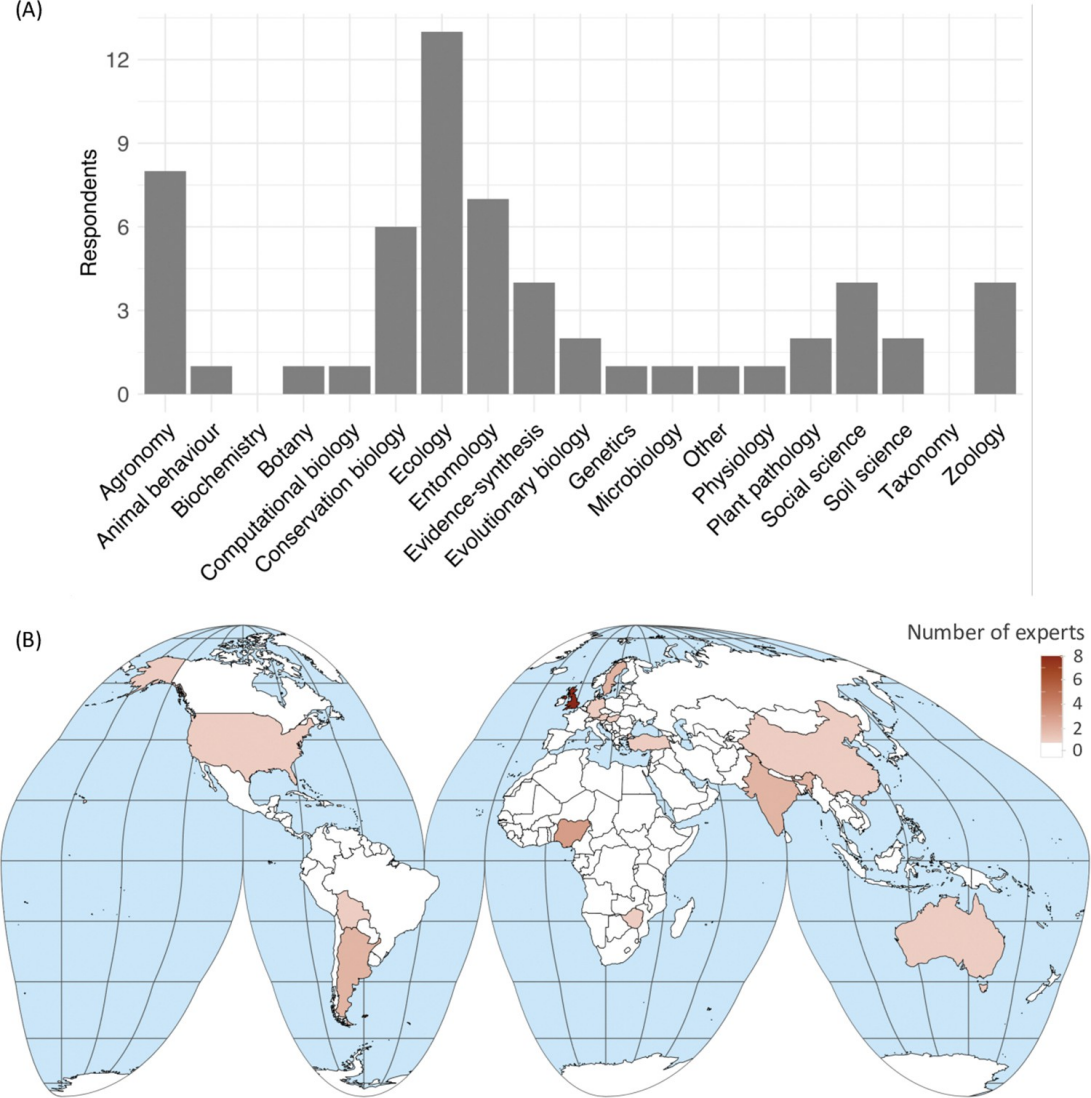
(A) A barchart showing the scientific fields that experts (n = 23) have worked in. These are the fields that we identified as relevant for this study, rather than a comprehensive list of all scientific fields. The “Other” scientific field denotes a free text field that one responded used to fill in the field “Agroecology and Ecosystem Service Management”. (B) A world map using Goode’s Homolosine projection, which accurately shows country sizes. Latitude and longitude are shown in grey lines and countries are outlined in black. Brown colour shows the number of experts (n = 23) with affiliations in each country. Team members could select multiple countries and scientific fields. The map was produced using the R packages *Tidyverse* [46], *rworldmap* [47] and *Simple Features* [48].

The final team represented a range of scientific expertise (Fig 1(A)) and academic career stages (number of experts: 0 years: 1, 1–5 years: 3, 6–10 years: 4, 11–20 years: 9, 21–30 years: 1, 31+ years: 5) in academia. There was a bias towards the fields of ecology and agronomy, which was intended given the study topic (Fig 1(A)). We had experts in arable (14 experts), agroforestry (7), pastoral (5), and mixed (3) systems, with experience working in temperate (14), tropical (11), subtropical (8) and polar and subpolar (1) regions (users could choose multiple answers for these questions). Team members were affiliated with institutions from across the world (Fig 1(B)), but there was a bias towards the UK (34% from the UK), where the study originated. The team included fifteen men and eight women.

### Developing AgroEcoList

First, three experts (co-authors) defined the scope of AgroEcoList, and drew up an initial list including 60 variables in the categories: experimental/sampling set-up, study site, and farm management (S2 in S1 File). We excluded variables related to sampling methods, statistical analysis, author composition, and funding because these require separate expertise to develop, and several guidelines and checklists already exist for these topics (e.g. [49–52]. We used this initial list to solicit feedback from the wider team via an online survey, where respondents were asked to rate each variable from one (“irrelevant”) to seven (“essential”), with the additional option of “not sure”. In the initial list, we aimed to include the most important variables for reporting in ecological studies in agriculture in addition to some “red herrings” (e.g. moon phase) to capture the full range of ratings in the survey. We included open text boxes to solicit further comments and suggestions. We used the results of this survey to direct focus-group discussions at four online workshops with the experts. We developed the checklist iteratively; in the first workshop, we discussed the results of the survey and drew up a new checklist; in the second workshop, we discussed the checklist developed in the first workshop, etc. Then, we user-tested AgroEcoList with three researchers who were not involved in the checklist development: Dr Michael Pashkevich, University of Cambridge; Dr Michael Garratt, University of Reading; and Dr Maxime Aeraerts, Washington State University. We used their feedback to refine the checklist and guidelines.

### Community survey

We conducted an online survey to get feedback from the wider community and to learn about their perceptions of reporting guidelines and reporting standards in agroecology. We chose an online survey method as we needed wide-ranging feedback from across the world with a large sample size. The structure was similar to the survey conducted by O’Dea *et*. *al* (2021) [19] when developing the PRISMAEcoEvo reporting guidelines. The survey questions were broken down into three sections (questions provided in full in S3 in S1 File). Most questions had options for “Not sure”, “N/A”, or “other” as appropriate. Questions were not randomised and, with the exception of the acceptance of the terms and conditions, were not mandatory. We asked respondents about:

1. Their experiences and expertise working in agroecology. This was to identify biases in the sample. These included questions about: their country/countries of affiliation, the climatic zone(s) that their research was based in (e.g. temperate or tropical), the system(s) that their research focused on (e.g. arable or pastoral), the scientific field(s) that describes their research, and their experience publishing agroecological research (e.g. author or editor).
2. Their knowledge and opinions of reporting guidelines, including: whether they had heard of reporting guidelines and (if they had) whether they thought they improved reporting standards and how they had used reporting guidelines before (e.g. as an author or reviewer). We asked them to rate on a scale of 1 (very poor) to 5 (excellent) the current reporting standards in agroecological studies for: experimental/sampling set-up (e.g. number of replicates), study site description (excluding soil—e.g. co-ordinates), soil description (e.g. soil type), livestock management (e.g. livestock species), crop and grassland management (e.g. fertiliser type), and outputs (e.g. crop yield).
3. Their perceptions of AgroEcoList. We gave a brief overview and asked: whether they would use the list as authors, reviewers, or editors; whether they had any additional comments; and to provide their emails if they would like to be updated.

We ran the survey from 22^nd^ March to 12^th^ April 2022 following approval from the University of Reading’s ethics committee [reference number: 1668D]. Co-authors promoted it via: social media (e.g. twitter); emails to colleagues; and departmental and society email lists and workspaces related to agriculture, ecology, or open science (*British Ecological Society’s (BES) Agricultural Ecology Special Interest Group; ReproducibiliTea; Society for Open*, *Reliable*, *and Transparent Ecology and Evolutionary Biology (SORTEE); The Ecological Society of America*). We reviewed survey responses at the end of each week and targeted regions with low responses using our wide-ranging network of collaborators. Based on these reviews, we also targeted journal editors by emailing Senior Editors from some relevant journals (*Agriculture*, *Ecosystems*, *and Environment; Agronomy for Sustainable Development; Basic & Applied Ecology; Ecology Letters; Journal of Agricultural Science;* and *Journal of Applied Ecology*). Co-authors did not answer the survey themselves. The final checklist was reviewed by all experts again following the user testing and community survey.

### Analysis

Open-ended responses were coded into themes by manual sorting. Data were visually analysed using packages *Tidyverse* [46], *rworldmap* [47], *Simple Features* [48], *nVennR* [53], *rsvg* [54], *grImport2* [55], and *cowplot* [56] in R version 4.1.1 [57] with R studio version 1.4.1717 [58].

## Results

### Checklist following expert consultation

The checklist following expert consultation consisted of 42 variables. It was different from the initial checklist both in structure and content (SI 3). It was more comprehensive and broader in scope, e.g. it included production outputs, which are relevant in productive landscapes. A summary plot of the survey ratings which were used to inform discussions, and the additional variables proposed in the survey and in the discussions can be seen in S1 Fig and S4 in S1 File. *(Note*, *this is not the final checklist*, *which is provided below*.*)*

### Community survey

**Fig 2:**
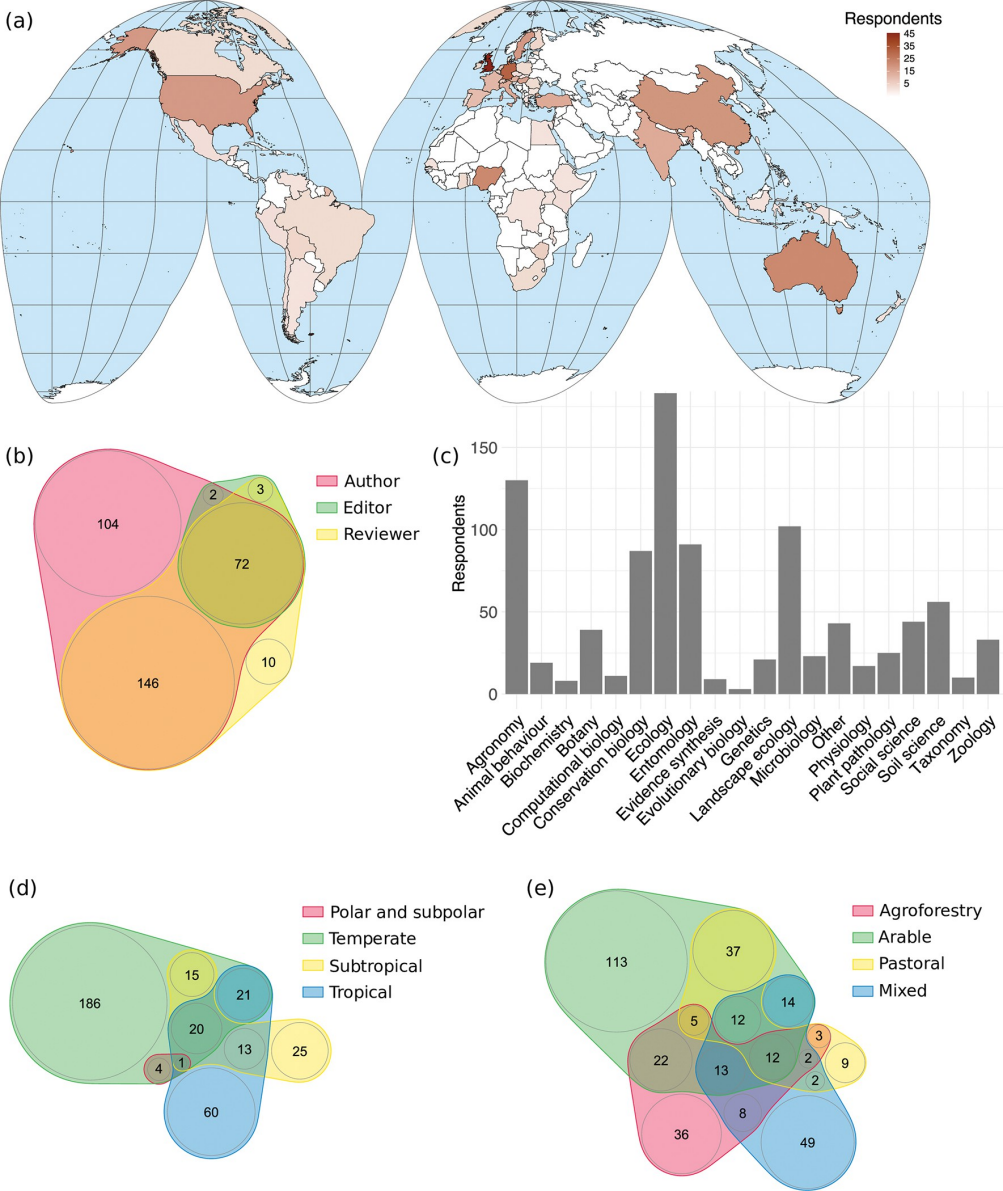
A composite figure showing the expertise and experience of respondents to the wider-community survey (n = 345). (a) A world map using Goode’s Homolosine projection, which accurately shows country sizes. Latitude and longitude are shown in grey lines and countries are outlined in black. Brown colour shows the number of respondents with affiliations in each country. (b,d,e) Venn diagrams showing how many respondents have: (b) published, reviewed, or edited agroecological research; (d) conducted research in polar and subpolar, temperate, subtropical or tropical regions; (e) conducted research in agroforestry, arable, pastoral or mixed agricultural systems. Circle areas (area) show the number of respondents in each category, which is written in black. Overlapping circles indicate cases where respondents chose multiple categories. (c) A barchart showing the scientific fields that respondents have worked in. These are the fields that we identified as relevant for this study, rather than a comprehensive list of all scientific fields. Respondents could select multiple answers to all questions. The map was produced using the R packages *Tidyverse* [46], *rworldmap* [47] and *Simple Features* [48].

Four hundred and nineteen people responded the survey, but only 345 responses completed section 1 and were included in our analyses. Questions in section one (experience and expertise of respondents) had a high response rate (99.6%). Respondents spanned a range of academic career stages (0 years: 7, 1–5 years: 73, 6–10 years: 85, 11–20 years: 97, 21–30 years: 60, 31+ years: 21) and 53 countries of affiliation (Fig 2(A)). There were regional biases, including high response rates from the United Kingdom (13%, *n* = 46), Germany (9%, *n* = 31), Nigeria (6.4%, *n* = 22), Australia (6.4%, *n* = 22), and China (5.5%, *n* = 19) (Fig 2(A)). This survey also showed that 93.3% of respondents had authored papers on agroecology, 67% had reviewed them, and 22.3% had edited them (Fig 2(B)). This followed the expected pattern as editors are a subset of reviewers, and reviewers tend to be a subset of authors. We had respondents with expertise across the breadth of scientific fields, with a bias towards Ecology (53%) and Agronomy (37.7%), which was expected given the study topic (Fig 2(C)). In terms of latitudinal ecoregions, 33.3% of respondents worked in tropical regions, 21.5% in subtropical regions, 71.6% in temperate regions, and 1.4% in polar and subpolar regions (Fig 2(D)). In terms of ecosystems, 29.3% of respondents worked in agroforestry, 66.1% worked in arable, 23.8% worked in pastoral, and 32.5% worked in mixed systems (Fig 2(E)). The low response rate from polar and subpolar regions is unlikely to indicate a bias as agricultural systems in these regions are currently limited [59]. However, these results show a high sample of respondents working in temperate regions and arable systems, which may have biased the results.

**Fig 3:**
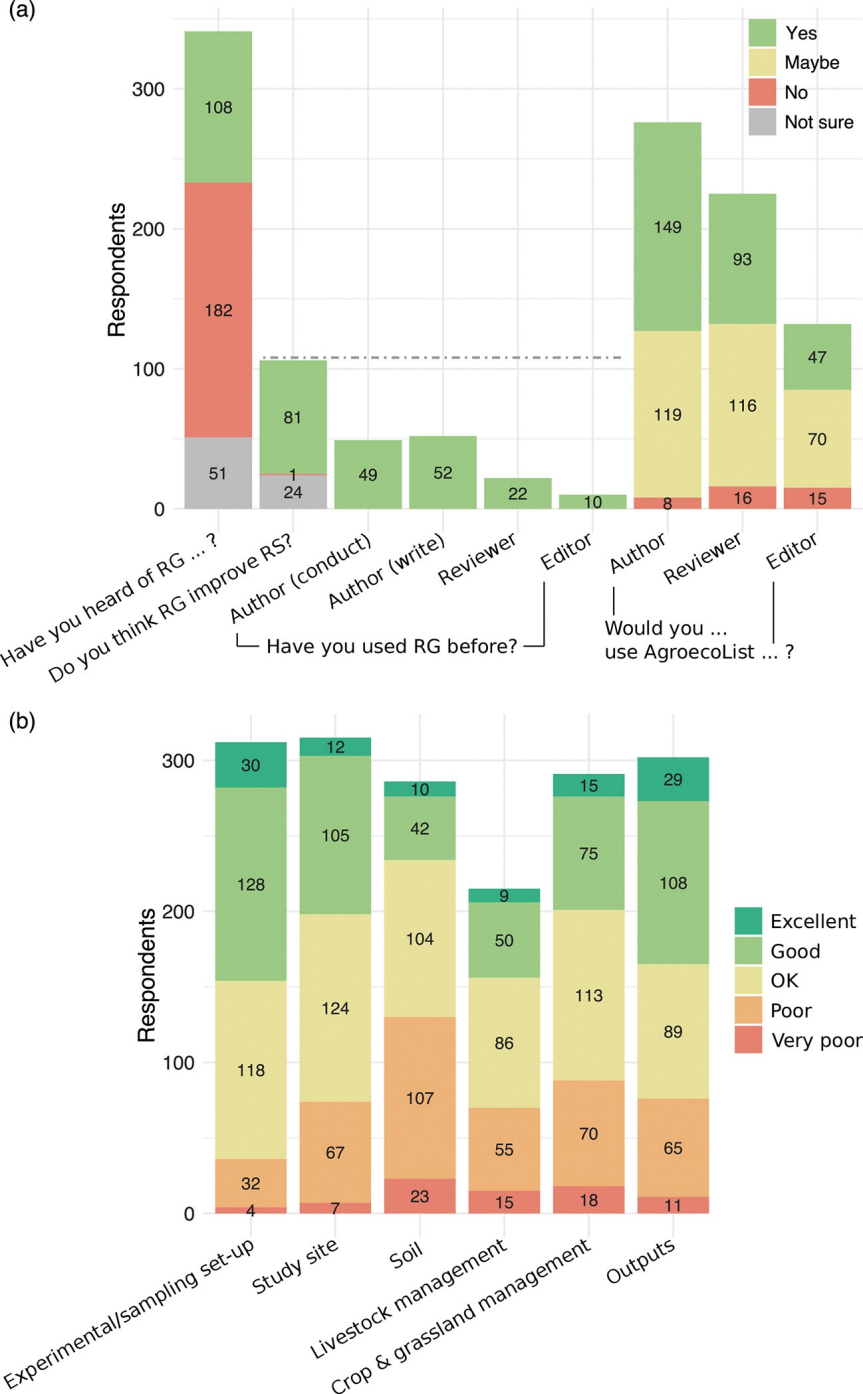
Barcharts showing respondents (n = 345) answers to questions about reporting guidelines (RG) and reporting standards (RS) via an online survey. Colours show responses and numbers indicate number of respondents. Questions were optional, and some respondents did not answer all questions. (a) Dashed grey line indicates the maximum number of respondents that answered these questions (108), as respondents were only asked this question if they answered “yes” to having heard of reporting guidelines before. (b) Shows responses to the statement “Rate the current standard of reporting in agroecological studies.”; the bars follow the order in the legend, with excellent at the top and very poor at the bottom. Questions are given in full in SI 4 in S1 File.

In section two, response rates were high for the questions on respondents’ knowledge and opinions of reporting guidelines (98.8%). The majority of respondents had not heard of (53%) or were not sure whether they had heard of (15%) reporting guidelines prior to completing the survey (Fig 3(A)). Of the respondents that had heard of reporting guidelines, 76.4% thought that they improved reporting standards, 22.6% were not sure, and 1% thought that they did not improve reporting standards (Fig 3(A)). Respondents had used reporting guidelines as authors to write (45%) or conduct (48%) studies, as reviewers (20%), and/or as editors (9%) (Fig 3).

Response rates were lower for the section two question asking respondents to rate reporting standards in agroecological studies (83%). This may be due to respondents not feeling knowledgeable enough to answer the question, as response rates dipped in a middle category (Livestock– 62%) and increased in a later one (Outputs– 88%) (Fig 3(B)). Averaged over all categories, respondents thought that reporting standards in agroecological studies were OK (36.8%), good (29.5%), poor (23%), excellent (6.1%) or very poor (4.5%). Perception of reporting standards was not consistent between categories, with experimental set-up having the highest perceived reporting standards (50.6% excellent or good and 11.5% very poor or poor) and soil the lowest (18.2% and 45.5% respectively) (Fig 3(B)).

Questions in section three (their opinions on AgroEcoList) had lower response rates (61.2%), potentially due to survey fatigue and/or the questions not being relevant (e.g. respondents may have skipped questions about whether they would use AgroEcoList as an editor if they were not editors). The majority of respondents indicated that they would use AgroEcoList as authors (54.0% yes, 43.1% maybe, 2.9% no), whereas the majority indicated that they might use it as reviewers (41.3%, 51.6%, 7.1%) or editors (35.6%, 53%, 11.4%) (Fig 3(A)). More respondents stated that they would use AgroEcoList as authors, reviewers, and editors, than the number that said they had used reporting guidelines already (158 vs 82).

Twenty-four percent of respondents (*n* = 83) commented in the open text box asking for feedback on reporting standards or AgroEcoList. We manually coded these into three themes “*General Comments”*, “*Suggested changes*”, “*Concern about implementation*” with 13 subthemes which are described below (see S1 Text for all responses). Three comments spanned multiple themes.

**General comments** consisted of two subthemes:

1. **Positive comments (no action needed)** (*n* = 20) were comments that solely indicated support for AgroEcoList and did not require any action, e.g. “*Great initiative and very helpful …*”.
2. **Other (no action needed)** (*n* = 9) were comments without a clear action, e.g. *“If it can be used*, *then*, *convenience and easy going is important”*.

**Suggested changes** consisted of six subthemes:

1. **AgroEcoList scope** (*n* = 4). Prior to the survey, we presented AgroEcoList as a checklist for agroecological research. Several respondents noted issues with this as “agroecology” can have multiple meanings and we had not included social variables, e.g. “*If it is a topic related to agroecology I am missing different social aspects …*”. We changed the definition to “ecological research in agriculture” in response to this.
2. **Variables already included** (*n* = 10) were suggestions for variables that were already in the list, e.g. “… *rotation*, *use of multicropping …”*. We clarified the variable definitions.
3. **Variables out of scope** (*n* = 7) were suggestions for variables that were beyond the scope of AgroEcoList, e.g. “*… sharing standards for code and data analysis …*”. We clarified the scope. We made one exception and added a note on collection methods for soil and recommend users report soil depth as several respondents noted that it is important for putting soil measurements in context.
4. **Variables adapted/added** (*n* = 5) were suggestions for variables that we added or adapted. We added a variable for cost/profitability as management decisions in agricultural landscapes are influenced by finances [60]: “… *what about something related to costs/profitability… if we are aiming to make decisions about practices…*”. We rearranged the “Livestock” section as some existing variables were overlapping: “… *density and rotations are dependent on each other*. *Maybe better to include grazing management type …”*
5. **Variables not added** (*n* = 13). We did not add the remaining suggested variables, many of which were outputs (e.g. “… *aboveground biomass …*”), which we think will not be relevant in enough cases to justify lengthening the list.
6. **Future developments** were suggestions to develop a standard metadata format (*n* = 1) and implement AgroEcoList at a journal level by recommending it to authors, reviewers, and editors (*n* = 2). We support these ideas as future developments of AgroEcoList.

**Concern about implementation** consisted of four subthemes:

1. **Privacy** (*n* = 4). Several respondents raised privacy concerns for landowners due to reporting co-ordinates, e.g. “*Co-ordinates … can often be linked to a specific farm …”*. We added a note to the bottom of AgroEcoList to highlight this risk to users.
2. **Collecting all data** (*n* = 10) were concerns about time/resource limitations for reporting the variables, e.g. “*… it can be tedious to report all the situations*.”. We clarified that AgroEcoList does not require all variables to be reported, it is a suggested minimum checklist.
3. **Stemming creativity** (*n* = 3) were concerns about the inflexibility of standard formats, e.g. “*These are important but over regulation and inflexible requirements can also be harmful to the scientific writing and reviewing process*.”. We agree that this could become an issue if users are overburdened with lists, but we argue that guidelines are currently needed to address low reporting standards [6].

### Final checklist and guidelines for use

The final checklist consisted of 42 variables in seven groups: experimental/sampling set-up, study site, soil, livestock management, crop and grassland management, outputs, and finances (Table 1). AgroEcoList 1.0 is a reporting checklist for ecology studies in agriculture. Readers can download it as a spreadsheet in S1 Checklist, with unique variable codes, detailed explanations of what to include for each variable, three completed examples [61–63]. It is also available on github (https://github.com/AgroecoList/Agroecolist), and users are advised to check this repository for updated versions and further examples. This repository will be maintained by Rothamsted Research, UK.

**Table 1:** AgroEcoList 1.0.

Variable Type	Variable Name
Experimental/ sampling set-up	Start & end date of study
	Start & end dates of any interventions/treatments
	Dates/frequency that measurements were taken
	Size & shape of experimental units (e.g. subplot/plot/field/farm)
	Experimental/sampling designs (e.g. blocked/randomised, distance between plots)
	Number of replicates
Study Site	Co-ordinates & co-ordinate system (e.g. WGS84)^a^
	Country
	Site map
	Elevation
	Slope & aspect
	Weather during study period
	Extreme/atypical events (e.g. flooding, fire, pest outbreak)
	Farm/crop/livestock certification/scheme (e.g. Organic)
	Artificial structure (e.g. open vs polytunnel, barn vs field)
	Landscape context (e.g. field-edge management, proximity to forest)
	Previous land use type & timing of transition (e.g. if converted from forest)
Soil^b^	Soil type & soil system (e.g. USDA)
	Soil texture (silt, sand, clay)
	Soil pH
	Soil organic matter
Livestock management	Livestock species & breed (including managed pollinators)
	Livestock grazing management (including density, timing, rotations)
	Livestock feeding regime (e.g. free grazing/supplemental)
	Livestock agrochemicals type, rate & timing (list all) (including medicines)
	Livestock demography (e.g. lifestage/sex)
Crop & grassland management	Crop species & variety (including main crops, secondary crops & non-cash-crops)
	Crop planting density & arrangement (e.g. broadcast/inter-row)
	Crop planting/harvesting timing (including rotations)
	Cultivation method, depth, & timing
	Mowing/topping method, height & timing
	Weeding method & timing
	Physical control of animal pests (e.g. trapping) method & timing
	Biological control agent species, release rate & timing
	Irrigation method, rate & timing
	Fertiliser type, rate & timing (list all)
	Crop protection chemicals type, rate & timing (list all)
	Other chemicals type, rate & timing (list all)
Outputs	Yield
	Quality and/or Commercial grade
Finances^c^	Any available costs/profits (e.g. interventions, management)

See the main text for user guidelines. Readers can download AgroEcoList as a spreadsheet in S1 Checklist, with unique variable codes, detailed explanations of what to include for each variable, and three completed examples.

^a^ Please ensure that you are not sharing sensitive information that can identify individuals or organisations without their permission (e.g. use a low resolution)

^b^ Sampling methods are beyond the scope of AgroEcoList, but we want to highlight the importance of reporting sampling depth when reporting soil variables

^c^Please ensure that you are not sharing confidential information.

AgroEcoList 1.0 can be used in three ways:

1. As a guide for the data to consider collecting when planning or conducting a study
2. As a memory aid to authors, reviewers, or editors for the variables that are relevant in ecology studies in agriculture.
3. As a template to fill in and be included in a table format in publications. This could be included in the supplementary information. We recommend that AgroEcoList is used as a template where possible, as this could facilitate data extraction, and therefore reuse and synthesis, which are essential in all scientific fields.

Collecting all of the variables in AgroEcoList 1.0 represents a significant body of work for authors, and in many cases authors will not have information for every variable. **Missing values (“Not recorded”) are expected, and should be accepted by readers, reviewers and editors.** The list is to encourage the inclusion of variables that are easy to report and may have been otherwise excluded, it should not be used to overburden authors by requesting additional information that is difficult to add. The aim is to encourage better reporting, rather than penalise authors that have not followed a specific format. For example, reviewers or editors could recommend the checklist to authors in cases where the study is not clearly reported, but if the relevant metadata are included in another format (e.g. archived as open-access datasheets) then we do not recommend delaying publication to reformat the information. Ideally authors would be made aware of relevant checklists prior to their initial submission (e.g. via author guidelines), and even prior to planning their study so they can consider the breadth of metadata that may be relevant.

Some of the variables in the list are more ambiguous (e.g. landscape context) than others (e.g. number of replicates). We have loosely-defined the parameters of some variables as strictly defining them would be too restrictive; we recommend that authors define these variables within the context of their study while also considering the potential contexts within which their study may be reused. AgroEcoList 1.0 should be revised and updated as necessary; reporting guidelines and the relevance of the variables listed here is likely to change through time. It should be used in tandem to standardised metadata formats [38, 40–42].

## Discussion

AgroEcoList 1.0 is a reporting checklist with 42 variables for ecological studies in agriculture. It can be used as a guide for authors, reviewers, and editors, and to provide a template for preparing publications. The checklist is to encourage the inclusion of variables that are easy to report and may have been otherwise excluded, such as reporting “zero” practices (e.g. “no herbicide was applied”). We do not recommend that reporting variables is made mandatory, and the list should not be used to overburden authors by requesting additional information that is difficult to add (see Guidelines for Use above). AgroEcoList should be used in tandem to standardised metadata formats that aim to facilitate data reuse and automated data extraction, e.g. ICASA Data Standards for data from agricultural field experiments [41, 42], DarwinCore for taxonomic data [38], and AgrO for agronomic data [40]. As with any reporting guidelines, AgroEcoList 1.0 should be revised as standards and the relevance of these variables will change; and users should check the online repository for updates (https://github.com/AgroecoList/Agroecolist). AgroEcoList was created with the aim of improving reporting standards in ecological studies in agriculture, following a similar strategy to that which has proved successful in medical research [15–17]. Improving reporting standards reduces research wastage [64], which will facilitates data reuse, for example for syntheses, which are a crucial for apply science to practice through evidence-based management or policy making [65]. By improving the description of the context within which the research was conducted (e.g. the soil type that the study was conducted in), practitioners can also better evaluate the relevance of research to their specific situation. Better reporting standards also benefits individual researchers as publications with more complete reporting are likely to receive more citations [5].

The wider community survey indicated that many respondents saw the need for AgroEcoList, and most would use it as authors, reviewers, and/or editors, despite many not having used reporting guidelines before. The majority of respondents (72%) thought that reporting standards in agroecology were OK, good, or excellent, with higher perceived standards in some categories (e.g. Experimental set-up) than others (e.g. Soil). However, these perceived standards may not reflect reporting standards in reality, as a community survey of evidence syntheses in ecology and evolutionary research indicated that respondents overestimate reporting standards [19]. In fact, current reporting standards are often poor, with basic details regularly missing from reports [6–8, 10], and reporting guidelines (such as AgroEcoList) can improve reporting standards [15–19].

Some survey respondents recommended that reporting guidelines are implemented by journals, e.g. that they are recommended to authors, reviewers and editors via author guidelines, and indeed some journals already do this (e.g. the *International Journal of Ecology* (Published by *Hindawi)* (Hindawi, 2022), *Agricultural Research (Springer)* (Springer, 2022), and the *Journal of the Science of Food and Agriculture (Wiley)* (Wiley, 2022)). We support this suggestion, and including reporting guidelines in author guidelines has been shown to improve reporting standards in other fields [15, 17, 18]. Better promotion is crucial, as our survey indicated that the majority of respondents had not or were not sure whether they had (68%) heard of reporting guidelines. However, of those that had heard of them, the majority (76%) thought that they improved reporting standards (23% were not sure), which shows that they are perceived as having a beneficial impact among those that are aware of them. Curating reporting guidelines via an online, open-access platform similar to the EQUATOR Network [21], which is a database of reporting checklists for medical research, could increase the uptake of reporting guidelines agriculture and ecology. This study can act as a blueprint to guide the development of more reporting checklists in the biological sciences [19, 26–29]. In particular, we recommend a community-driven approach that engages the wider community [19].

## Supporting information

S1 Checklist Spreadsheet version of checklist with variable explanations and three examples. (XLSX)

S1 File Includes: S1 Expert survey questions, S2 Initial vs final checklist, S3 Wider community survey questions, S4 Suggested variables from survey and workshops, S1 Fig. Expert survey variable rating.
(PDF)

S1 Text Open text comments from community survey. (XLSX)
