# Supplementary material for: AgroEcoList 1.0: A checklist to improve reporting standards in ecological research in agriculture
Source: PLoS One. 2023 Jun 13;18(6):e0285478. doi: 10.1371/journal.pone.0285478 (PMC10263310; doi:10.1371/journal.pone.0285478)

SUPPLEMENTARY INFORMATION FOR: AgroEcoList 1.0: A checklist to improve reporting standards in ecological research in agriculture

Georgia M. Daykin, Marcelo A. Aizen, Luke Barrett, Lewis Bartlett, Péter Batáry, Lucas A. Garibaldi, Ali Guncan, Sridhar Gutam, Bea Maas, Jayalakshmi Mitnala, Flavia Montaño-Centellas, Tarirai Muoni, Erik Öckinger, Ode Okechalu, Richard Ostler, Simon Potts, David C. Rose, Cairistiona F. E. Topp, Hope O. Usieta, Obaiya G. Utoblo, Christine Watson, Yi Zou, William J. Sutherland, Amelia S. C. Hood*

*Corresponding author email: a.s.hood@reading.ac.uk

CONTENTS

S1 Expert survey questions

S2 Initial vs final checklist

S3 Wider community survey questions

S4 Suggested variables from survey and workshops

S1 Fig. Expert survey variable rating

ADDITIONAL SUPPLEMENTARY INFORMATION PROVIDED SEPARATELY

S5 Open text comments from community survey

S6 Spreadsheet version of checklist with variable explanations and three examples

SI 1. Expert survey questions

The following questions followed an introductory page which described the project and how the data would be stored and processed. Personal identifiers (e.g. name and email address) were not collected (i.e. the data were anonymised). Questions and options in multiple choice questions were presented in the same order. Questions 1-8 were compulsory. For questions 1-4 respondents could select multiple answers. We obtained ethical approval from the University of Reading to conduct this survey. Answer options are given in italics.

Section 1 – Experiences and expertise

1. What is/are your country/countries of affiliation? *List of countries given to select from*

2. Which climatic zone(s) has your research been based in? *Tropical/Subtropical/Temperate/Polar and Subpolar*

3. Which system(s) has your research been on? *Arable/Pastoral/Agroforestry/Mixed*

4. Which scientific field(s) best describes your research (current and past)? This list is not comprehensive, and some fields are nested in each other (e.g. entomology in zoology), but we hope this will give an idea of the breadth of expertise in the team. *Agronomy/Animal behaviour/Biochemistry/Botany/Computational biology/Conservation biology/Ecology/ Entomology/Evidence-synthesis/Evolutionary biology/Genetics/Microbiology/Other (open text)/ Physiology/Plant pathology/Social science/Soil science/Taxonomy/Zoology*

5. How many years have you worked in agriculture? *0 years/1-5 years/6-10 years/11-20 years/21-30 years/31+ years*

6. How many years have you worked in academia? *0 years/1-5 years/6-10 years/11-20 years/21-30 years/31+ years*

7. What is your gender? *Male/female/non-binary/Prefer not to say/Other (open text)*

Section 2 – Checklist

8. There are seven sections to the checklist: (1) Location, (2) Timing, (3) Experimental set-up, (4) Finance, (5) Crops, (6) Land management, (7) Chemical applications
   Please rate the following variables in response to the question "How important is it to include information on [variable] in an agricultural study? Please note we are not include social science studies in this, so the focus is on ecological studies. This is because we think that the checklist for social science studies in agriculture would be quite different.
   Ratings: 1 (Irrelevant) – 2 – 3 – 4 (Sometimes important) – 5 – 6 – 7 (Essential) – Not sure
   Note: variables were listed as in the initial checklist shown in SI 3.

9. Are there any additional items which you think should be added to the checklist? *Open text*

10. Additional comments or suggestions: *Open text*

SI 2. A table showing the variables in the initial checklist vs the final checklist (AgroEcoList 1.0). Some variables in the initial checklist were added as "red herrings" (e.g. moon phase) to capture the full range of ratings in the survey (SI 1).

	INITIAL LIST
Location	Co-ordinates
	Specific location e.g., nearest town & county
	Country
	Climate type e.g., IUCN habitat classification scheme
	Soil type e.g., USDA
	Soil colour
	Elevation
	Plot aspect
	Average annual temperature
	Total annual rainfall
	Moon phase
	Artificial structure (whether the study was in a greenhouse, open field, polytunnel)
Timing	Start and end date of study
	Start and end date of any interventions
	Justification for duration
	Planting and harvesting date of crops
	Date that measurements were taken
	Date that fertiliser was purchased
Experimental set-up	Experimental design (e.g., randomised, controlled, correlated)
	Number of replicates
	Size of plots
	Distance between plots (control to control and control to treatment)
Finances	Cost of any interventions
	Cost of labour
	Estimated value of farm equipment
	Crop value
Crop management	Species and variety (main crop)
	Planting density (main crop)
	Species and variety (secondary crops)
	Planting density (secondary crops)
	Planting arrangement (e.g., inter-row, inter-strip)
	Crop rotation species
	Crop rotation timing
	Crop yield
Land management	Ploughing method
	Ploughing depth
	Ploughing timing
	Weeding method
	Weeding timing
	Biological control agent species
	Biological control agent abundance
	Biological control agent timing
	Grazer species
	Grazer colour
	Grazer abundance
	Grazer timing
	Irrigation method
	Irrigation rate
Chemical applications	Fertiliser type
	Fertiliser amount
	Fertiliser timing
	Herbicide type
	Herbicide amount
	Herbicide timing
	Insecticide type
	Insecticide amount
	Insecticide timing
	Fungicide type
	Fungicide amount
	Fungicide timing

	AGROECOLIST
Experimental/ sampling set-up	Start & end date of study
	Start & end dates of any interventions/treatments
	Dates/frequency that measurements were taken
	Size & shape of experimental units (e.g. subplot/plot/field/farm)
	Experimental/sampling designs (e.g. blocked/randomised, distance between plots)
	Number of replicates
Study Site	Co-ordinates & co-ordinate system (e.g. WGS84)
	Country
	Site map
	Elevation
	Slope
	Aspect
	Weather during study period
	Extreme/atypical events (e.g. flooding, fire, pest outbreak)
	Farm/crop/livestock certification/scheme (e.g. Organic)
	Artificial structure (e.g. open vs polytunnel, barn vs field)
	Landscape context (e.g. field-edge management, proximity to forest)
	Previous land use type & timing of transition (e.g. if converted from forest)
Soil	Soil type & soil system (e.g. USDA)
	Soil texture (silt, sand, clay)
	Soil pH
	Soil organic matter
Livestock management	Livestock species & breed (including managed pollinators)
	Livestock grazing management regime (including density, timing, rotations)
	Livestock feeding regime (e.g. free grazing/supplemental)
	Livestock agrochemicals type, rate & timing (list all) (including medicines)
	Livestock demography (e.g. lifestage/sex)
	Livestock management timing (e.g. release/rotations)
Crop & grassland management	Crop species & variety (including main crops, secondary crops & non-cash-crops)
	Crop planting density & arrangement (e.g. broadcast/inter-row)
	Crop planting/harvesting timing (including rotations)
	Cultivation method, depth, & timing
	Mowing/topping method, height & timing
	Weeding method & timing
	Physical control of animal pests (e.g. trapping) method & timing
	Biological control agent species, release rate & timing
	Irrigation method, rate & timing
	Fertiliser type, rate & timing (list all)
	Crop protection chemicals type, rate & timing (list all)
	Other chemicals type, rate & timing (list all)
Outputs	Yield
	Quality and/or Commercial grade
Finances	Any available costs/profits (e.g. interventions, management)

SI 3. Wider community survey questions

The following questions followed an introductory page which described the project and how the data would be stored and processed. The data were partially anonymous; respondents could provide their email addresses at the end of the survey if they wanted to be kept up to date on future developments with AgroecoList. Contact details were stored separately to the other survey responses. Questions and options in multiple choice questions were presented in the same order. Questions were not compulsory. Questions 9 and 10 were only asked if the answer to question 8 was "yes". We obtained ethical approval from the University of Reading to conduct this survey. Answer options are given in italics. The survey structure was similar to the survey conducted by O'Dea *et. al* (2021) (29) when developing the PRISMAEcoEvo reporting guidelines. *Respondents could select multiple answers.

Section 1 – Experiences and expertise

1. What is/are your current country/countries of affiliation? **List of countries given to select from*

2. Which climatic zone(s) has your research been based in? **Tropical/Subtropical/Temperate/Polar and Subpolar*

3. Which system(s) has your research been on? **Arable/Pastoral/Agroforestry/Mixed*

4. Which scientific field(s) best describes your research (current and past)? **Agronomy/Animal behaviour/Biochemistry/Botany/Computational biology/Conservation biology/Ecology/ Entomology/Evidence-synthesis/Evolutionary biology/Genetics/Landscape ecology/Microbiology/Other (open text)/ Physiology/Plant pathology/Social science/Soil science/Taxonomy/Zoology*

5. How many years have you worked in agriculture (including research, industry, and practice)? *0 years/1-5 years/6-10 years/11-20 years/21-30 years/31+ years*

6. How many years have you worked in academia (including PhD)? *0 years/1-5 years/6-10 years/11-20 years/21-30 years/31+ years*

7. What is your experience publishing agroecological research? **Author/Reviewer/Editor*

Section 2 – Knowledge and opinions of reporting guidelines

8. Had you heard of reporting guidelines prior to completing this survey? E.g. PRISMA (Preferred Reporting Items for Systematic Reviews and Meta-analyses), ROSES (RepOrting Standards for Systematic Evidence), TTEE (Tools for Transparency in Ecology and Evolution). *Yes/No/Not sure*

9. Do you think reporting guidelines improve reporting standards? *Yes/No/Not sure*

10. Have you used reporting guidelines before? **Yes: as an author, to help conduct a study/Yes: as an author, to help write a study/Yes: as a reviewer, to assess manuscripts/Yes: as an editor, to assess manuscripts/Yes: other (please specify)/No/Not sure*

11. Rate the current standard of reporting in agroecological studies. Options: Experimental/sampling set-up (e.g. number of replicates, size of plots); Study site description (excluding soil) (e.g. elevation, site map); Soil description (e.g. soil type); Livestock management (e.g. livestock species); Crop & grassland management (e.g. fertiliser type); Outputs (e.g. crop yield). *Ratings: 1 – very poor/2 – poor/3 – ok/4 – good/5 – excellent/don't know*

Section 3 – Opinions of AgroecoList

AgroecoList is a reporting checklist for agroecological studies. Reporting checklists are known to improve reporting standards, and are increasingly recommended in journal guidelines. AgroecoList was developed by a team of 23 researchers.

AgroecoList can be used in two ways:
1. As a memory aide to authors, reviewers, or editors
2. As a template to fill in and be included in a table format in publications, e.g. in supplementary information. This would facilitate automated data extraction via machine learning, which would promote data reuse, such as synthesis.

Collecting all of the variables in AgroecoList represents a significant body of work, and in many cases authors will not have information for every variable. Missing values ("Not recorded") are expected, and should be accepted by readers, reviewers and editors. The list is to encourage the inclusion of variables that are easy to report and may have been otherwise excluded, it should not be used to overburden authors by requesting additional information that is not easy to add and critical for the original study.

Some of the variables are more ambiguous than others. We have loosely-defined the parameters of some variables as strictly defining them would be too restrictive; we recommend that authors define these variables within the context of their study while also considering the potential contexts within which their study may be reused.

Then we provided a table with AgroecoList and a llink to a spreadsheet with detailed explanations of each variable

12. Would you or your students use AgroecoList for your next agroecological study? Options: Author, Reviewer, Editor. **Yes/Maybe/No/NA*

13. Any other comments on reporting standards in agroecology or AgroecoList: *Open text*

14. If you would like to be updated when AgroecoList is available for use, please leave your email below: *Open text*

SI 4. Suggested variables from the survey and workshops with 23 experts. Many of these variables were included in the final list either in their original form (green ✓) or they were adapted (green ✓). Several were excluded for being beyond the scope of the checklist (orange ⊗), for being too obvious (i.e. something that is nearly always recorded) (orange ⊗) or for being difficult to collect and relevant in a few cases (orange ⊗). These decisions were made during four online workshops and email exchanges with the 23 experts.

Initial list group: experimental set-up

- ✓ Within-field sampling design
- ✓ Field size
- ✓ Farm size
- ⊗ Details of statistical analysis
- ⊗ Aim of interventions

Initial list group: location

- ✓ Landscape context
- ✓ Map or google earth kml file of sample sites
- ✓ Soil chemical properties
- ✓ History of land use
- ⊗ Weather data collection duration and equipment
- ⊗ Climatic conditions of previous year

Initial list group: finances

- ⊗ Funders of study
- ⊗ Economics of experiment (e.g., cost of intervention vs profits)

S1 Fig. Scatterplot showing the average survey rating (point) and standard error (bar) for each of the proposed checklist variables following the online survey with 21 experts. Ratings run from 1 (irrelevant) to 7 (essential). Variables are coloured according to the variable group from the initial checklist (SI 3) and ordered from largest to lowest average rating.

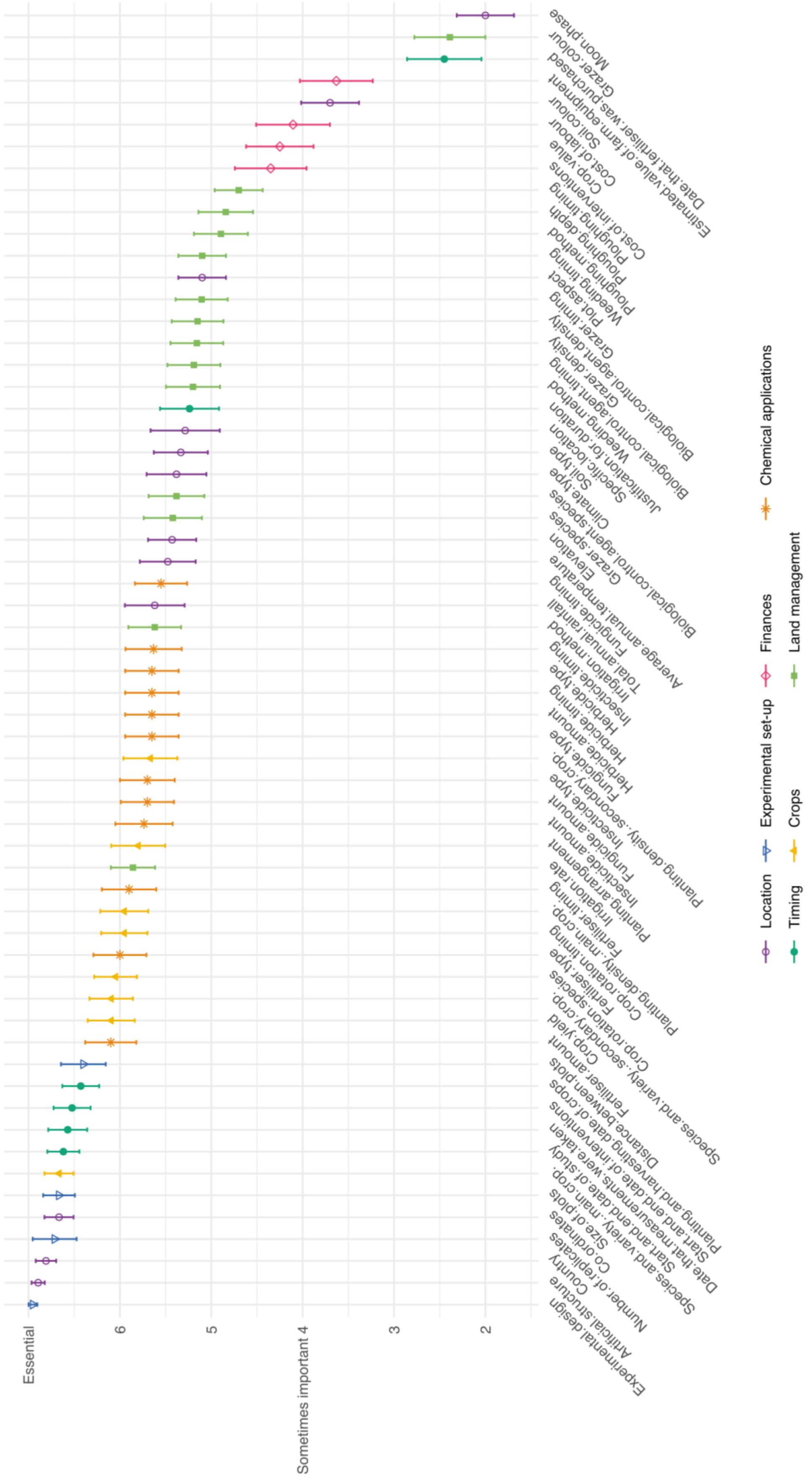

Supplement: S1 File — Expert survey variable rating. (PDF) [file pone.0285478.s002.pdf]
